# Two cases of carbonic anhydrase VA deficiency—An ultrarare metabolic decompensation syndrome presenting with hyperammonemia, lactic acidosis, ketonuria, and good clinical outcome

**DOI:** 10.1002/jmd2.12171

**Published:** 2020-10-01

**Authors:** Ashish Marwaha, Judy Ibrahim, Taylor Rice, Nadia Hamwi, Charles Anthony Rupar, David Cresswell, Chitra Prasad, Andreas Schulze

**Affiliations:** ^1^ Clinical and Metabolic Genetics The Hospital for Sick Children Toronto Ontario Canada; ^2^ Department of Academic Affairs Tawam Hospital Al Ain Abu Dhabi United Arab Emirates; ^3^ Schulich School of Medicine & Dentistry London Ontario Canada; ^4^ Department of Biochemistry Western University London Ontario Canada; ^5^ Department of Pathology and Laboratory Medicine Western University London Ontario Canada; ^6^ Department of Pediatrics Grand River Hospital Kitchener Ontario Canada; ^7^ Department of Pediatrics Western University London Ontario Canada; ^8^ Department of Pediatrics University of Toronto Toronto Ontario Canada; ^9^ Department of Biochemistry University of Toronto Toronto Ontario Canada

**Keywords:** encephalopathy, ketonuria, lactic acidosis, metabolic acidosis, neonatal hyperammonemia

## Abstract

The combination of neonatal hyperammonemia, lactic acidosis, ketonuria, and hypoglycemia is pathognomonic for carbonic anhydrase VA (CA‐VA) deficiency. We present two cases of this rare inborn error of metabolism. Both newborns with South Asian ancestry presented with a metabolic decompensation characterized by hyperammonemia, lactic acidosis and ketonuria; one also had hypoglycemia. Standard metabolic investigations (plasma amino acids, acylcarnitine profile, and urine organic acids) were not indicative of a specific organic aciduria or fatty acid oxidation defect but had some overlapping features with a urea cycle disorder (elevated glutamine, orotic acid, and low arginine). Hyperammonemia was treated initially with nitrogen scavenger therapy and carglumic acid. One patient required hemodialysis. Both have had a favorable long‐term prognosis after their initial metabolic decompensation. Genetic testing confirmed the diagnosis of carbonic anhydrase VA (CA‐VA) deficiency due to biallelic pathogenic variants in *CA5A*. These cases are in line with 15 cases previously described in the literature, making the phenotypic presentation pathognomonic for this ultrarare (potentially underdiagnosed) inborn error of metabolism with a good prognosis.

## 1 INTRODUCTION

**FIGURE 1:**
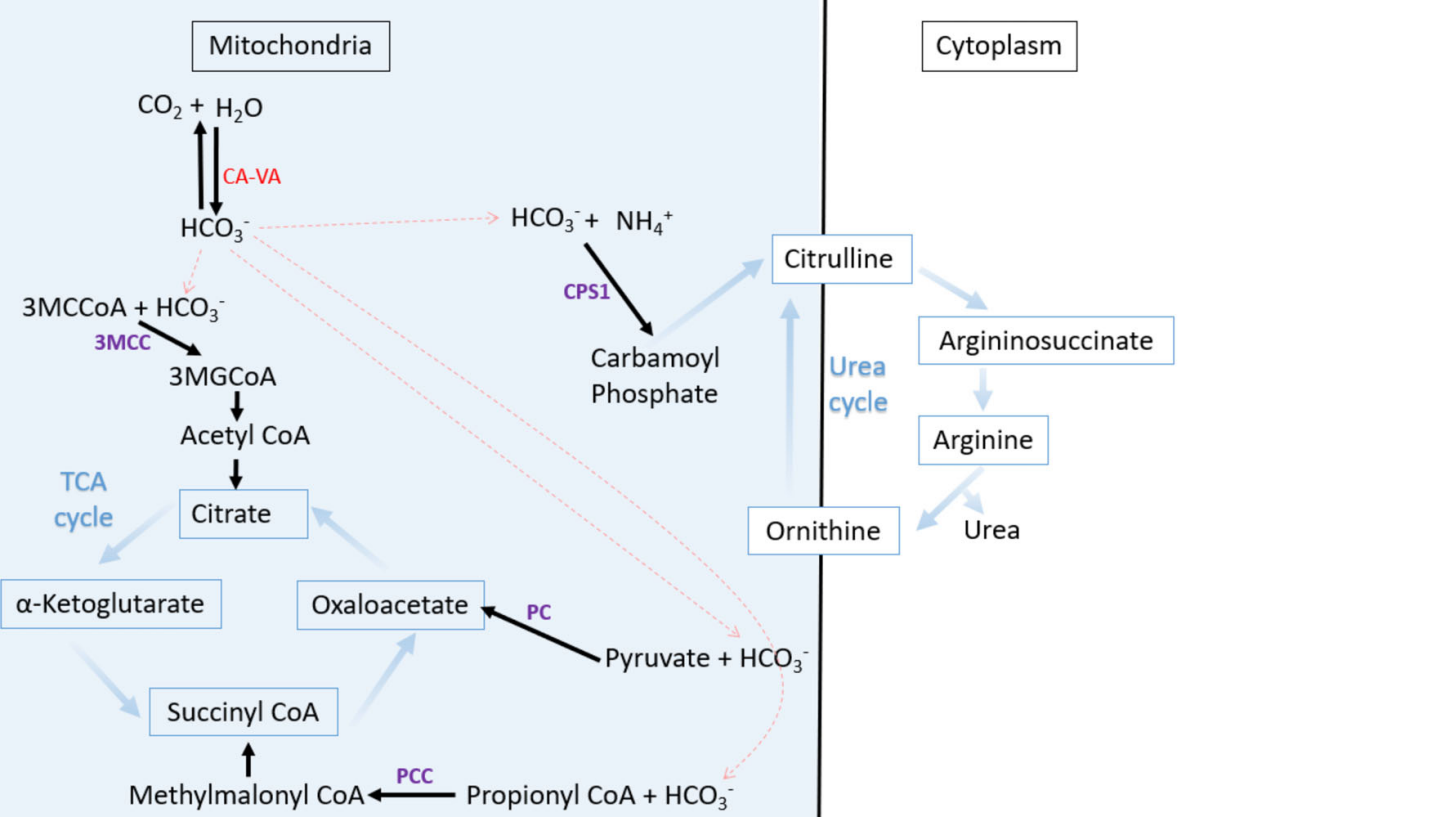
The intermediary metabolism of carbonic anhydrase (CA‐VA). Carbonic anhydrases catalyze the reversible hydration of carbon dioxide and produce bicarbonate. Human carbonic anhydrase VA (CA‐VA) is a mitochondrial enzyme present in the liver, kidney, and skeletal muscle. CA‐VA supplies bicarbonate as a substrate of four mitochondrial enzymes: carbamoyl phosphate synthetase 1 (CPS1), 3‐methylcrotonyl‐CoA carboxylase (3MCC), propionyl‐CoA carboxylase (PCC), and pyruvate carboxylase (PC)

Carbonic anhydrases catalyze the reversible hydration of carbon dioxide and produce bicarbonate. Human carbonic anhydrase VA (CA‐VA) is a mitochondrial enzyme present in the liver, kidney, and skeletal muscle. CA‐VA supplies bicarbonate as a substrate of four mitochondrial enzymes: carbamoyl phosphate synthetase, 1,3‐methylcrotonyl‐CoA carboxylase 1, propionyl‐CoA carboxylase, and pyruvate carboxylase (Figure 1). Biallelic variants in *CA5A* cause a rare autosomal recessive inborn error of metabolism called CA‐VA deficiency (OMIM 615751). Patients with this condition have metabolic crises, which are characterized by encephalopathy, hyperammonemia, lactic acidosis, ketonuria, and hypoglycemia. The first report of CA‐VA deficiency identified four patients from two unrelated families.^1^ In 2016, a larger case series of 10 patients was reported.^2^ Subsequent to these initial reports, only one further case has been reported in the literature.^3^ We describe here two cases of CA‐VA deficiency who have a similar presentation to the 15 cases previously reported in the literature. Our cases both presented with metabolic acidosis, high lactate and hyperammonemia and responded well to standard therapy with a good prognosis following the initial metabolic decompensation.

## 2 CASE 1

The first case is a male infant who was born to nonconsanguineous parents originally from Punjab, India. The baby was delivered at term by caesarian section after a failed trial of induction of labor and presence of maternal fever. He was born with APGAR scores 9^1^ and 9^5^ and his birth weight was 4250 g (87th centile).

At day 1 of life, he was noted to be jittery and had two episodes of hypoglycemia recorded at 1.7 and 1.9 mmol/L (>2.4 mmol/L). These episodes corrected promptly with feeds. He became very irritable the following day and developed tachypnea and tachycardia. Blood gas analysis showed pH of 7.57 (7.35‐7.45), PCO_2_ < 18 mmHg (35‐45), HCO_3_^−^ of 13.2 mmol/L (22‐28) with base excess of −5.1 (−2 to +3) indicating mixed metabolic acidosis and respiratory alkalosis. Blood lactate was 7.5 mmol/L (0.9‐2.4) and ammonia was 254 μmol/L (15‐55). He was started on intravenous fluids, antibiotics (ampicillin and gentamycin), sedated, intubated, and transferred to a tertiary care center with a suspicion of an inborn error of metabolism. Metabolic investigations, as shown in Table 1, were not diagnostic of a urea cycle disorder or organic aciduria. The metabolites found in the urine organic acids raised the consideration of multiple carboxylase deficiency, though biotinidase deficiency was ruled out with enzymatic testing.

**TABLE 1:** Summary of initial metabolic investigations in the two cases

Metabolic investigation	Case 1	Case 2	
Newborn screening	Normal	Normal	
Plasma amino acids, carnitine (μmol/L)	*Day 2 of life*: Elevated alanine 788 (70‐387) Elevated glutamine 949 (66‐620) Low arginine 16 (22‐78) Normal citrulline 17 (10‐38) Low free carnitine 4 (10‐30) Normal total carnitine 27 (15‐45)	*Day 5 of life*: Elevated alanine 1130 (325‐425) Normal arginine 39 (30‐70) Normal citrulline 15 (0‐26) Low free carnitine 3.5 (12‐60) Low total carnitine 9.5 (23‐84) *Day 7 of life*: High free carnitine 494 (12‐60) High total carnitine 526 (23‐84)	
Urine orotic acid (mmol/mol creatinine)	Slightly elevated 6 (0.0‐5.6)	Normal 1.9 (1.4‐5.3)	
Urine organic acids	*Day 2 of life*: Lactic acid (large) Ketones (large) Dicarboxylic acids(large) Pyruvic acid (large) 3‐Hydroxyisovaleric acid (large) 2‐Hydroxy‐3‐methylsuccinic acid (large) 3‐Methylcrotonylglycine (small) Tiglylglycine (small) 2‐Ethyl‐3‐hydroxypropionic acid (small) Palmitic acid (small) *Days 3, 17, 51 of life*: Unremarkable	*Day 5 of life*: Lactic acid Ketones Dicarboxylic acids Liver metabolites (large) 2‐Hydroxyisovaleric acid (small) 3‐Hydroxyisovaleric acid (small) 3‐Hydroxy‐3‐methylglutaric acid (small) 2‐Ethyl‐3‐hydroxypropionic acid (small)	
Acylcarnitine profile (μmol/L)	*Day 2 of life*: C4‐OH 0.61 (<0.33) C6‐OH 0.18 (<0.04) Day 51 of life: C2 39.8 (7.6‐27.8) C3 0.97 (<0.65) C6‐OH 1.23 (>0.16)	*Day 5 of life*: Initially low C0 11.6 C2 7.35 C3 0.82 C4 0.06 C8:1 0.08 C8 0.08 C10:1 0.02 C5DC 0.05 C12:1 0.03 C12 0.07 C120H 0.02 C14:2 0.09 C14:1 0.03 C14 0.16	*Day 7 of life*: After carnitine Supplement Large multiple Elevations noted 488 (18‐47) 33.2 (4.6‐35.4) 2.53 (<1.08) 1.31 (<0.68) 1.26 (<0.68) 0.64 (<0.30) 0.68 (<0.29) 0.38 (<0.14) 0.27 (<0.19) 0.21 (<0.19) 0.22 (<0.05) 0.27 (<0.09) 0.23 (<0.21) 0.10 (<0.11)
*CA5A* sequencing	Allele 1: c.721G>A, p.(Glu241Lys) Allele 2: c.619‐?_774+?del	Allele 1: c.721G>A, p.(Glu241Lys) Allele 2: c.721G>A, p.(Glu241Lys)	

At 56 hours of age, the hyperammonemia was initially managed with intravenous nitrogen scavengers (sodium benzoate/sodium phenylacetate), L‐arginine, dextrose and lipids. After a slight initial decline, his ammonia level increased to 361 μmol/L within 4 hours and he became nonresponsive. Hemodialysis was initiated which reduced the ammonia level to 64 μmol/L. Post hemodialysis, he was given carglumic acid 100 mg OD and managed with biotin 5 mg OD until biotinidase deficiency was ruled out.

A brain MRI at 4 days of life was unremarkable. The blood ammonia level remained <100 μmol/L and the baby was discharged at 9 days of life with no dietary restrictions. Subsequent ammonia levels, growth and development have remained normal. He is currently 19 months old, growing well, and meeting all developmental milestones without receiving medications. The family has been provided with a medical care plan in event of any decompensation. At 45d of age, a hyperammonemia targeted gene panel identified two likely pathogenic heterozygous variants in *CA5A*: c.721G>A, p.(Glu241Lys), and c.619‐?_774+?del. The two variants were confirmed by Sanger sequencing and qPCR, respectively. Parental follow‐up testing showed the variants were in *trans*.

## 3 CASE 2

The second case is a 9‐year‐old boy who was born at term to consanguineous parents (first cousins) of Sri‐Lankan descent. He was delivered at term by spontaneous vaginal delivery. He was born with APGAR scores 8^1^ and 9^5^ and his birth weight was 3065 g (25th centile). The pregnancy was complicated only by gestational diabetes.

After initial discharge home on day 2, he was readmitted to the neonatal unit at 72 hours of age due to poor feeding, jaundice, tachycardia, tachypnoea, and lethargy. Laboratory investigations revealed a significant metabolic acidosis pH of 7.16 (7.35‐7.45), PCO_2_ mmHg 13 (35‐45), HCO_3_^−^ of 5 mmol/L (22‐28) with base excess of −21 (−2 to +3), and an anion gap of 33 (<15). He also had an elevated lactate at 6.6 mmol/L (0.9‐2.4) and ammonia at 363 μmol/L (15‐55). He was resuscitated with fluid boluses, given a bicarbonate correction, started on antibiotics and transferred to a tertiary center intensive care unit for ongoing management. His initial metabolic investigations are shown in Table 1. The results were not thought to be diagnostic of a particular condition, though, multiple carboxylase deficiency, pyruvate carboxylase deficiency and mitochondrial disorder were high in the differential diagnosis. His initial plasma carnitine levels were below the normal range (total carnitine 9.5 μmol/L [23‐84] and free carnitine 3.5 μmol/L [12‐60]). A repeat acylcarnitine profile, after intravenous carnitine supplementation, showed multiple elevations. That along with the presence of multicystic kidneys led to the suspicion of a potential diagnosis of multiple acyl‐CoA dehydrogenase deficiency (glutaric aciduria type 2, GA2). However, sequencing of *ETFA*, *ETFB*, and *ETFDH* genes was negative and fibroblast analysis revealed a normal palmitate oxidation acylcarnitine profile (WJ Rhead, Wisconsin, USA). Mitochondrial fibroblast studies showed normal results for pyruvate dehydrogenase, pyruvate carboxylase, 2‐oxoglutarate dehydrogenase, cytochrome c oxidase, succinate cytochrome c reductase, mitochondrial NADH cytochrome c reductase, oligomycin sensitive ATPase, citrate synthase, and cellular lactate/pyruvate ratio (B Robinson, Toronto, Canada). We therefore cannot explain the multiple elevations on the acylcarnitine profile, which may represent the consequence of intravenous carnitine supplementation.

Hyperammonaemia was managed with Ammonul (sodium benzoate, sodium phenylacetate), l‐carnitine, carglumic acid, l‐arginine, and dextrose. His ammonia returned to normal levels within 24 hours and he did not require hemodialysis. He was discharged on oral carnitine supplementation that was discontinued when he was 3 months old, and riboflavin 100 mg per day that was later increased to 150 mg per day and eventually discontinued at 3½ years of age. He underwent an MRI of the brain, which showed laminar subdural hematomas in the parietooccipital regions, posterior fossa, and posterior falx. There was also some nonspecific hyperintensity of the deep white matter regions in the frontal, parietal, and occipital lobes. He also had an abdominal ultrasound that showed small dysplastic and multicystic kidneys bilaterally.

Currently he is 9 years‐old and doing well clinically with no developmental concerns. He was issued with an emergency care plan to perform a blood gas, lactate, urinalysis for ketones, CK, glucose, transaminase, urea, creatinine, and electrolytes during any acute illness. However, he has not had any further decompensations. He eventually underwent targeted genetic sequencing of *CA5A*, which showed he was homozygous for the likely pathogenic variant c.721G>A, p.(Glu241Lys). His parents were confirmed to be heterozygous carriers for the variant.

## 4 DISCUSSION

Neonatal hyperammonemia with or without lactic acidosis has a wide differential diagnosis and often initial evaluation and management is targeted to a potential urea cycle disorder or organic aciduria. CA‐VA deficiency is an extremely rare, or perhaps underdiagnosed cause of hyperammonemia, which has the potential to lead to diagnostic confusion with other inborn errors of metabolism. The differential diagnosis of CA‐VA deficiency includes transient hyperammonemia of the newborn, pyruvate carboxylase deficiency, multiple carboxylase deficiency, CPS1 deficiency, and NAGS deficiency. The results of the metabolic investigations allow the differentiation of CA‐VA deficiency from above disorders. In transient hyperammonemia of the newborn, there is isolated hyperammonemia with all other metabolites often being normal (including glutamine and alanine). In pyruvate carboxylase deficiency there is an elevated citrulline and lysine, and proximal early infantile onset urea cycle defects typically have normal lactate and ketone levels and low plasma citrulline concentrations. In our patients, other diagnoses that were initially considered included multiple carboxylase deficiencies, multiple acyl‐CoA dehydrogenase deficiency, and mitochondrial disorders. The overlap with metabolites identified in multiple carboxylase deficiency (ie, holocarboxylase synthase deficiency or biotinidase deficiency) occurs as biotin is an obligatory cofactor for the three carboxylases that also require bicarbonate as a substrate. However, multiple carboxylase deficiency does not often present with hyperammonemia, and biotinidase deficiency was ruled out enzymatically in patient 1. Multiple acyl‐CoA dehydrogenase deficiency was considered because patient 2 had multicystic kidneys and a suggestive acylcarnitine profile after carnitine supplementation.

The two cases we describe here showcase the major presentation of CA‐VA deficiency that has been described previously.^1^, ^2^ The age of onset for both our cases was in the first few days of life. Previous reports have indicated an age of onset of between 2 days to 4 years but 67% of patients presented before day 5 of life. This early presentation indicates that newborn screening results may not be available at the time of presentation. Case 1 and case 2 were negative in the newborn screening test. All CA‐VA deficiency patients have presented with hyperammonemia (238‐1150 μmol/L), elevated lactate (4.8‐15 mmol/L) and ketonuria, and most had hypoglycemia as well. Our cases also had these essential features of the initial presentation. Metabolic acidosis is not a universal feature but was present in both our cases. In case 1, the metabolic profile was consistent with a possible urea cycle disorder, but this was not shown in case 2. The metabolites identified in the urine organic acid analysis of our cases are like those described in the 15 published CA‐VA deficient patients.^1^, ^2^ The main treatment is carglumic acid (Carbaglu), which is an analogue of N‐acetylglutamate (NAG) approved for treating acute hyperammonemia in NAGS deficiency.^4^, ^5^ Propionyl CoA carboxylase cannot function efficiently because of the HCO_3_^−^ deficiency caused by CA‐VA deficiency and propionyl CoA is a potent inhibitor of NAGS. Carbamoyl phosphate synthetase CPS1 does not function because of the NAG and HCO_3_^−^ deficiency. Carglumic acid has been shown to be beneficial in treating hyperammonemia in 53% of the published cases of CA‐VA deficiency.^2^ It was used as a therapy in both of our cases; however, case 1 needed hemodialysis. In our centers, carglumic acid is used as standard therapy during an unexplained first hyperammonemic episode. Due to the initial diagnostic uncertainty, other therapies more often used in other causes of hyperammonemia (nitrogen scavengers, arginine, lipids, and carnitine) were used initially in both cases. We also report a good neurodevelopmental outcome in our patients, which is consistent with 86% of the previously reported cohort. Ammonia levels, in both patients, have remained normal without nitrogen scavenger treatment, even during infections later in life. There have been no dietary modifications in either patient as they have been metabolically stable. More than 65% of patients with CA‐VA deficiency experienced a similar single neonatal hyperammonemia crisis without recurrence.^2^ It has been hypothesized that CA‐VA has an isoform, CA‐VB, whose expression is upregulated in the absence of functional CA‐VA later in life.^2^ There has been a recent single case report of fatality associated with CA‐VA deficiency at age 18 months, thus, it is important to monitor and measure ammonia promptly in events of any illnesses.^3^

Our patients were both from the Indian subcontinent as were 71% of the patients with CA‐VA deficiency previously identified. This propensity to identify cases from the same ethnic background raises the possibility of a founder effect. The gene *CA5A* is 50Kb long with seven exons and six introns. Most pathogenic variants occur in exons 4 and 6. The genetic variants in our cases have both been previously described. The c.721G>A, p.(Glu241Lys) (rs563971993) (NM_001739.2) variant was present in both cases and falls within exon 6. It has an overall population minor allele frequency of 3.09e−4 and has not been observed as homozygous before. This variant is observed in 0.27% of alleles in individuals from a south Asian background in the gnomAD database.^6^ The Glu241Lys change is a nonconservative amino acid substitution, which is likely to impact secondary protein structure as these residues differ in polarity, charge, size and/or other properties. This variant is reported as likely pathogenic variant in ClinVar database. The c.619–?_774+?deletion removes exon 6 and is similar to pathogenic deletions identified in ClinVar that have been reported in patients having Indian subcontinent ancestry.^2^

In summary, we present two cases which add to the limited previous reports of CA‐VA deficiency. CA‐VA deficiency is a recently described ultrarare inborn error of metabolism with most patients being from the Indian subcontinent. It presents early in life with a hyperammonemia crisis accompanied by respiratory alkalosis/metabolic acidosis, lactic acidosis, ketonuria, and hypoglycemia. The outcome is often favorable with normal neurodevelopment and no further crises following prompt and temporary treatment of hyperammonemia with carglumic acid.

## CONFLICT OF INTEREST

All authors have no conflict of interest to declare.

## References

[1] van Karnebeek CD , Sly WS , Ross CJ , et al. Mitochondrial carbonic anhydrase VA deficiency resulting from CA5A alterations presents with hyperammonemia in early childhood. Am J Hum Genet. 2014;94:453‐461. 24530203 10.1016/j.ajhg.2014.01.006 PMC3951944

[2] Diez‐Fernandez C , Rufenacht V , Santra S , et al. Defective hepatic bicarbonate production due to carbonic anhydrase VA deficiency leads to early‐onset life‐threatening metabolic crisis. Genet Med. 2016;18:991‐1000. 26913920 10.1038/gim.2015.201

[3] Baertling F , Wagner M , Brunet T , et al. Fatal metabolic decompensation in carbonic anhydrase VA deficiency despite early treatment and control of hyperammonemia. Genet Med. 2020;22:654‐655. 31641285 10.1038/s41436-019-0677-9

[4] Haberle J . Carglumic acid for the treatment of N‐acetylglutamate synthase deficiency and acute hyperammonemia. Expert Rev Endocrinol Metab. 2012;7:263‐271. 30780843 10.1586/eem.12.17

[5] Haberle J , Rubio V . Hyperammonemias and related disorders In: Blau N, Duran M, Gibson KM, Dionisi‐Vici C, eds. Physician's Guide to the Diagnosis, Treatment, and Follow‐Up of Inherited Metabolic Diseases. Heidelberg, New York, Dordrecht, London: Springer‐Verlag; 2014:47‐62.

[6] Karczewski K , Fancioli L , Tiao G , et al. The mutational constraint spectrum quantified from variation in 141,456 humans. Nature. 2020;581:434‐443. 32461654 10.1038/s41586-020-2308-7 PMC7334197

